# Cocaine Reduces the Neuronal Population While Upregulating Dopamine D2-Receptor-Expressing Neurons in Brain Reward Regions: Sex-Effects

**DOI:** 10.3389/fphar.2021.624127

**Published:** 2021-04-12

**Authors:** Kevin Clare, Chelsea Pan, Gloria Kim, Kicheon Park, Juan Zhao, Nora D. Volkow, Zhicheng Lin, Congwu Du

**Affiliations:** ^1^ Department of Biomedical Engineering, Stony Brook University, Stony Brook, NY, United States; ^2^ Laboratory of Psychiatric Neurogenomics, Basic Neuroscience Division, McLean Hospital, Belmont, MA, United States; ^3^ National Institute on Drug Abuse, Bethesda, MD, United States

**Keywords:** cocaine, dopamine-2 receptor, dynorphin, enkephalin, neuroadaptation, sex differences

## Abstract

Addiction to cocaine is associated with dysfunction of the dopamine mesocortical system including impaired dopamine-2 receptor (D2r) signaling. However, the effects of chronic cocaine on neuronal adaptations in this system have not been systematically examined and data available is mostly from males. Here, we investigated changes in the total neuronal density and relative concentration of D2r-expressing neurons in the medial prefrontal cortex (mPFC), dorsal striatum (Dstr), nucleus accumbens (NAc), and ventral tegmental area (VTA) in both male and female mice passively exposed to cocaine for two weeks. In parallel experiments, we measured mRNA levels for *Drd2* and for opioid peptides (m*Penk* and m*Pdyn*). Through a combination of large field of view fluorescent imaging with BAC transgenic D2r-eGFP mice and immunostaining, we observed that cocaine exposed mice had a higher density of D2r-positive cells that was most prominent in mPFC and VTA and larger for females than for males. This occurred amidst an overall significant decrease in neuronal density (measured with NeuN) in both sexes. However, increases in *Drd2* mRNA levels with cocaine were only observed in mPFC and Dstr in females, which might reflect the limited sensitivity of the method. Our findings, which contrast with previous findings of cocaine-induced downregulation of D2r binding availability, could reflect a phenotypic shift in neurons that did not previously express *Drd2* and merits further investigation. Additionally, the neuronal loss particularly in mPFC with chronic cocaine might contribute to the cognitive impairments observed with cocaine use disorder.

## Introduction

Addiction is a chronic disease which manifests by compulsive drug seeking and use that is difficult to control despite harmful consequences (Koob and Volkow, 2016). The involvement of dopamine in drug reinforcement is well recognized, but its role in addiction is less clear (Uhl et al., 2002). Imaging studies in drug addicted individuals have shown that dopamine function is markedly disrupted as reflected by decreases in dopamine (DA) release and in DA D2 receptor (D2r) availability in striatum (Volkow and Fowler, 2000). The reduced striatal D2r availability is associated with reduced activity in the medial and ventral prefrontal cortex–regions involved in salience attribution and motivation, which could underlie the compulsiveness of drug taking in addiction (Jasinska et al., 2015; Porrino et al., 2016). In parallel, studies in laboratory animals have also documented the involvement of the dopamine mesocortical system in addiction, including the substantia nigra (SN) and ventral tegmental area (VTA) midbrain regions with their striatal targets to dorsal striatum (Dstr) and nucleus accumbens (NAc) and cortical targets to the medial prefrontal cortex (mPFC) (Koob and Volkow, 2010). Preclinical studies have also reported reduced D2r levels in Dstr and NAc along with evidence of neuronal loss in mPFC and striatal regions with chronic cocaine exposure (Stefański et al., 2007; George et al., 2008). However, the systematic analyses of changes in D2r and neuronal loss in the various regions of the DA mesocortical system with chronic cocaine exposure as a function of sex to our knowledge has not been investigated. This is relevant since women when compared to men transition faster from occasional to compulsive cocaine intake and those with cocaine use disorder experience more cravings, withdrawal symptoms, and worse outcomes (Griffin et al., 1989; Robbins et al., 1999; Van Etten and Anthony, 2001; Becker and Koob, 2016). Similarly, studies in laboratories animals have reported significant sex differences in the locomotor and rewarding effects of cocaine (Kokane and Perrotti, 2020).

Here we aimed to assess the changes in total neuronal density and in the density of D2r expressing neurons in mesocortical dopamine regions (Dstr, NAc, VTA, and PFC) after repeated cocaine exposure. For this purpose, we combined a transgenic mouse model of D2r-enhanced GFP (D2r-eGFP) with immunostaining and fluorescence microscopy, which allowed us to separately visualize and quantify D2r-expressing and non-D2r neurons (Gong et al., 2003). In a separate group of wild-type (WT) mice we assessed the effects of cocaine on mRNA levels of D2r (m*Drd2*) and proenkephalin (m*Penk*) genes, which are genes that are co-expressed in D2r expressing neurons in striatum. As control, we measured the prodynorphin (m*Pdyn*) gene, which in striatum is expressed in dopamine 1 (D1) receptor expressing neurons (Whitfield et al., 2015). We hypothesized that chronic cocaine would result in neuronal loss and loss of D2r expressing neurons in striatum and PFC in parallel with a reduction in the mRNA levels of *Drd2* and *Penk*. We anticipate that the effects would be greater in females than in male mice.

## Materials and Methods

### Animals and Drug Treatments

**FIGURE 1:**
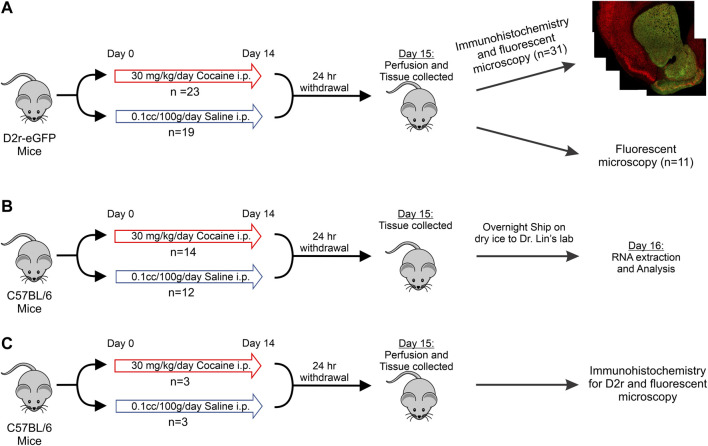
Schematic of the experimental protocol and timeline. **(A)**: D2r-eGFP tissue collection with immunohistochemistry for eGFP and NeuN or no immunohistochemistry for eGFP intensity quantification; **(B)**: C57BL/6 tissue collection and mRNA analysis; **(C)**: Drug treatment, immunohistochemistry, and imaging of Drd2 in C57BL/6 mice.

All experiments were approved by the Institutional Animal Care and Use Committee at Stony Brook University. The transgenic mouse strain, Tg(Drd2-eGFP)S118Gsat (in which eGFP was expressed under the *Drd2* promoter; D2r-eGFP (Gong et al., 2003)) as well as C57BL/6 WT animals were used. We studied 74 mice (age 4 months, males and females) comprised of two genotypes derived from C57BL/6 background, of whom 48 were used for immunohistochemistry and fluorescent microscopy imaging (Figures 1A,C; Table 1), and 26 for mRNA quantification (Figure 1B; Table 1). The mRNA and D2r immunostaining quantification studies were done in WT C57BL/6. All animals were kept on a reverse 12 h on/off light cycle with *ad libitum* access to food and water.

**TABLE 1:** Experimental Design: Animals, Pretreatment, Experimental approaches.

Animal	Pretreatment	Experimental metric	Groups (n)	Quantification	Brain regions analyzed
I: Controls (*n* = 34)	0.9% saline (0.2 cc/100 g/day, i.p.)	Fluorescence in Tg(Drd2-eGFP)S118Gsat	**1A)** No IHC (*n* = 5 ♂)	D2r fluorescence intensity	Dstr
			**1B)** GFP and Neun IHC (*n* = 14; 9♂ 5♀)	D2r cell and Neun counting	Dstr, VTA, NAc and mPFC
		mRNA in WT mice	**2)** rt-PCR (*n* = 12; 6♂ 6♀)	mRNA density	Dstr, VTA, NAc and mPFC
		D2r fluorescence in WT mice	**3) **D2r IHC (*n* = 3♂)	D2r cell counting	Dstr
II: Cocaine-animals (*n* = 40)	Cocaine (30 mg/kg/day, i.p.)	Fluorescence in Tg(Drd2-eGFP)S118Gsat	**4A)** No IHC (*n* = 6 ♂)	D2r fluorescence intensity	Dstr
			**4B)** GFP and Neun IHC (*n* = 17; 7♂ 10♀)	D2r cell and Neun counting	Dstr, NAc VTA, mPFC
		mRNA in WT mice	**5)** rt-PCR (*n* = 14; 7♂ 7♀)	mRNA density	Dstr, NAc VTA, mPFC
		D2r fluorescence in WT mice	**6)** D2r IHC (*n* = 3♂)	D2r cell counting	Dstr

The protocol of the drug pretreatment is illustrated in Figure 1. For all experiments, animals received the same treatment schedule: Cocaine mice received daily intraperitoneal (i.p.) injections of 30 mg/kg cocaine for 14 consecutive days followed by 24 h withdrawal prior to euthanizing. This dose of cocaine was chosen because previous studies had demonstrated its ability to recreate addiction behaviors and our imaging studies have shown that this protocol induced pathophysiological changes relevant to clinical adverse consequences from cocaine use including cerebral ischemia and transient ischemic attacks (Zombeck et al., 2009; Rappeneau et al., 2015; You et al., 2017). The control group was injected i.p. with 10cc/kg of saline daily following the same protocol as the cocaine treatment group.

### Immunohistochemistry

**FIGURE 2:**
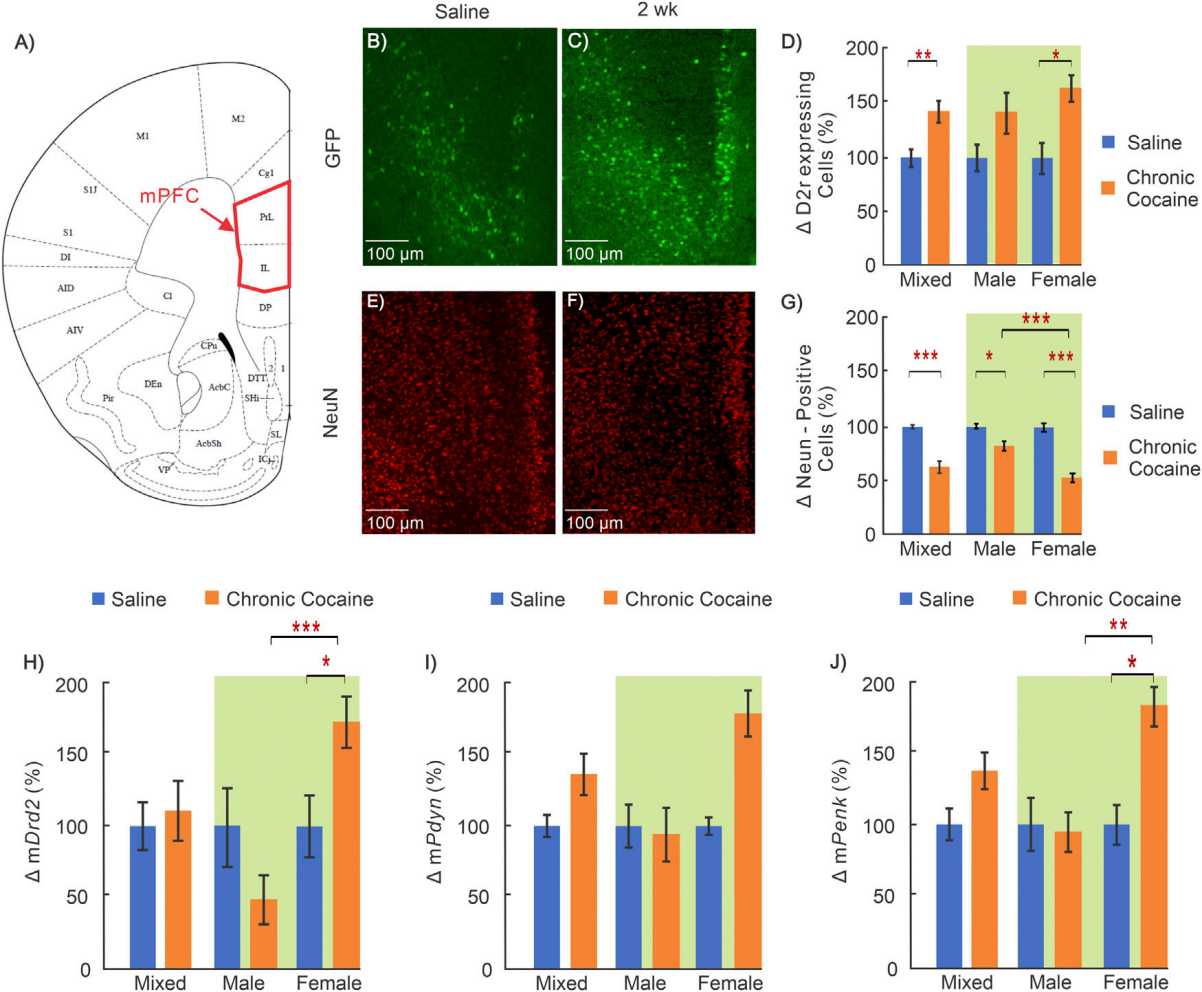
Cocaine effects in medial prefrontal cortex (mPFC). **(A)**: Mouse brain atlas showing the location of the microscopic images in mPFC (George and Franklin, 2001); **(B–C)**: “Zoom-in” GFP in mPFC of a saline and cocaine treated animal; **(D)**: Comparison of D2r-GFP expressing neurons in mPFC in saline (n = 14; 5 ♀, 9 ♂) and cocaine (n = 13; 6 ♀, 7 ♂) treated animals. **(E–F)**: Corresponding NeuN images in mPFC of a saline treated and cocaine treated mouse; **(G)**: Comparison of neurons in mPFC between saline (n = 10; 5 ♀, 5 ♂) and cocaine treated animals (n = 10; 6 ♀, 4 ♂); **(H–J)**: Comparison of mRNA levels between saline (n = 12; 6 ♀, 6 ♂) and cocaine (n = 14; 7 ♀, 7 ♂) treated WT mice for *Drd2* **(H)**, *Pdyn* **(I)**, and *Penk* **(J)**. Mixed: males and females; female: females only; male: males only; *: *p* < 0.05; **: *p* < 0.005; ***: *p* < 0.001. Blue bars correspond to saline and orange ones to cocaine treated mice. Green background indicates data included in two-way ANOVA. Values show averages and SE.

**FIGURE 3:**
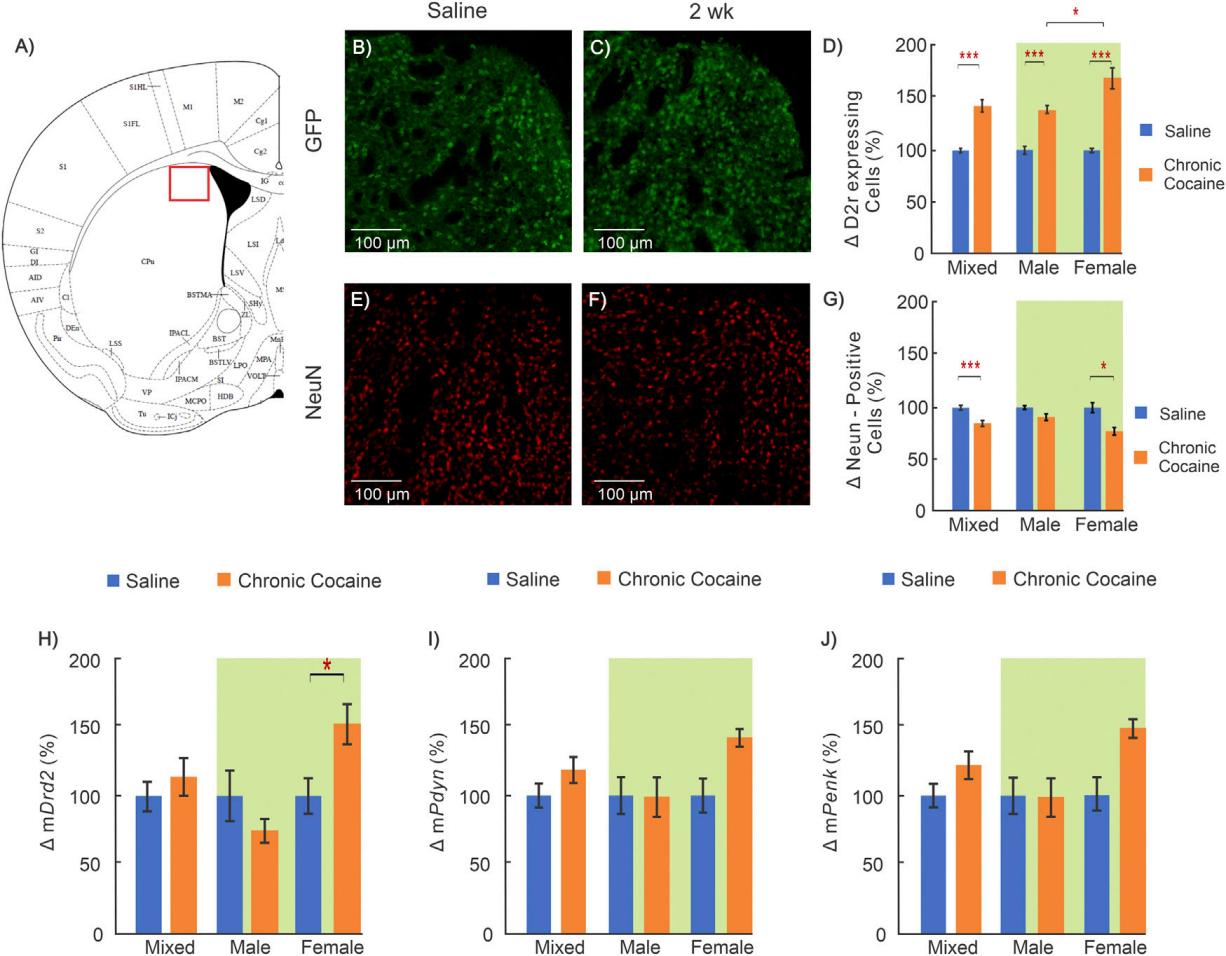
Cocaine effects in dorsal striatum (Dstr). **(A)**: Mouse brain atlas shows location where images were obtained for Dstr (George and Franklin, 2001) ; **(B-C)**: “Zoom-in” GFP images of a control and a cocaine exposed animal; **(D)**: Comparison of D2r-GFP expressing neurons in Dstr between saline (n = 12; 5 ♀, 7 ♂) and chronic cocaine (n = 11; 5 ♀, 6 ♂) exposed animals (2 weeks); **(E-F)**: Corresponding NeuN images of a saline treated and a cocaine exposed mouse; **(G)**: Comparison of neurons between controls (n = 11; 5 ♀, 6 ♂) and cocaine (n = 10; 6 ♀, 4 ♂) exposed animals; **(H-J)**: Comparison of mRNA levels between control (n = 12; 6 ♀, 6 ♂) and cocaine (n = 14; 7 ♀, 7 ♂) animals for *Drd2* **(H)**, for *Pdyn* **(I)** and *Penk* **(J)**. Mixed: males and females; female: females only; male: males only; *: *p* < 0.05; **: *p* < 0.005; ***: *p* < 0.001. Blue bars correspond to saline and orange to cocaine treated mice. Data for two-way ANOVA highlighted in green. Values show averages and SE.

**FIGURE 4:**
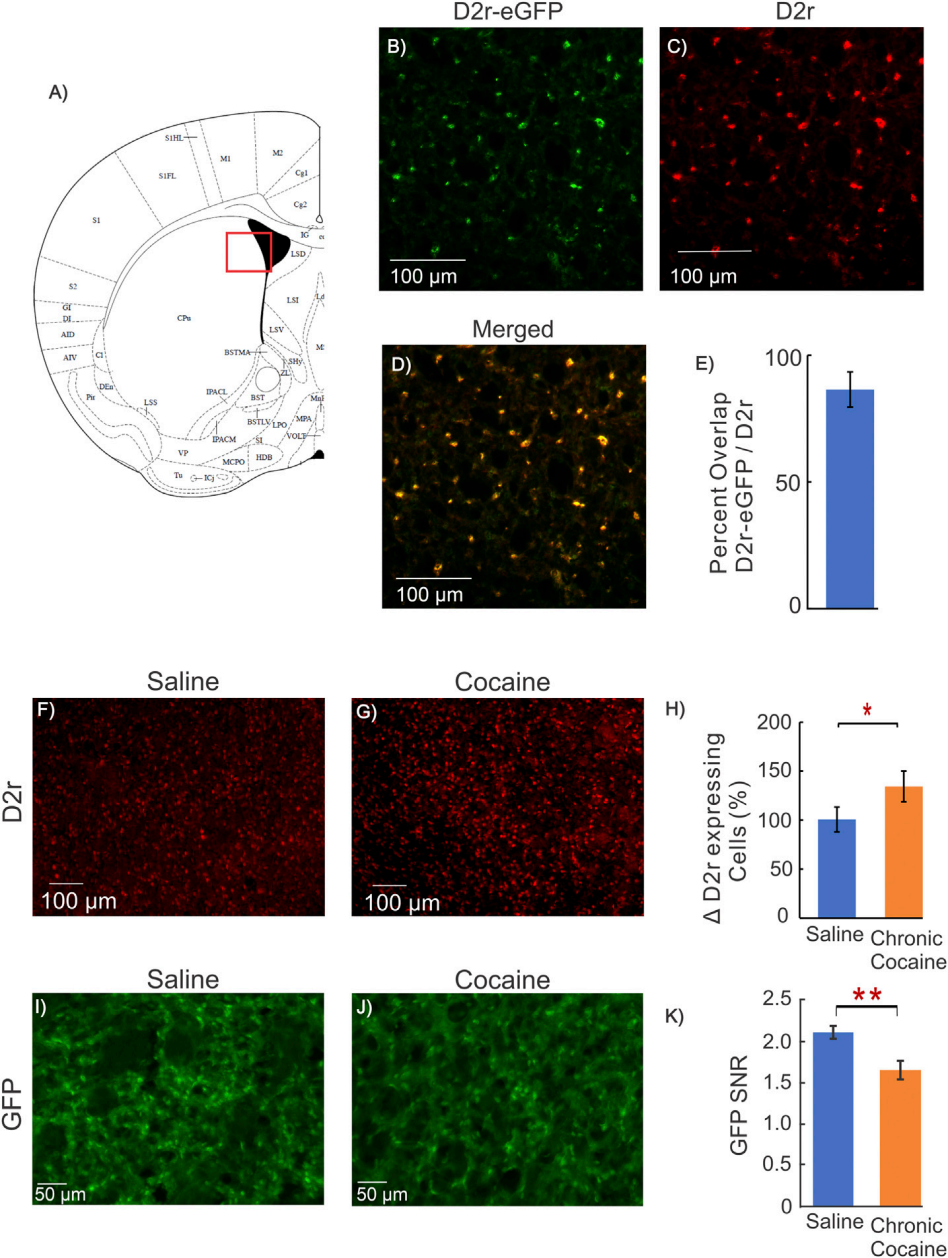
Dstr D2r Response in Cocaine WT mice and fluorescent intensity measure. **(A)**: Mouse brain atlas showing location of imaging in (George and Franklin, 2001); **(B–D)**: Representative images from a mouse brain of the co-localization of GFP from D2r-eGFP reporter line (green), D2r antibody (red), and merged (yellow) obtained from the dorsal striatum; **(E)**: Quantification of overlap of GFP and D2r antibody, indicating that GFP of the D2r-eGFP transgenic mouse reports cellular D2r expression; **(F, G)**: Representative images of D2r positive cell density in the dorsal striatum of a WT animal obtained using an antibody directed against D2r for a control (saline) and cocaine mouse; **(H)**: Quantification of the density of D2r-positive cells for 3 saline and 3 cocaine treated C57BL/6 male mice, indicating the D2r-neuronal expression increase after chronic cocaine exposure (p = 0.02); **(I–J)**: Representative images of *ex-vivo* GFP expression without immunohistochemistry from a control and a cocaine treated animal; **(K)**: Comparison of GFP Signal-to-Noise Ratio (SNR = GFP expression per D2r neuron/background noise) between control and cocaine treated mice. The GFP of D2r neurons in cocaine treated mice (1.66 ± 0.08, n = 6, m = 25) was significantly lower than in controls (2.11 ± 0.05, n = 5, m = 18, *p* = 0.002, Student t-test). Blue bars correspond to saline and orange bars to chronic cocaine. Values are means and SE.

**FIGURE 5:**
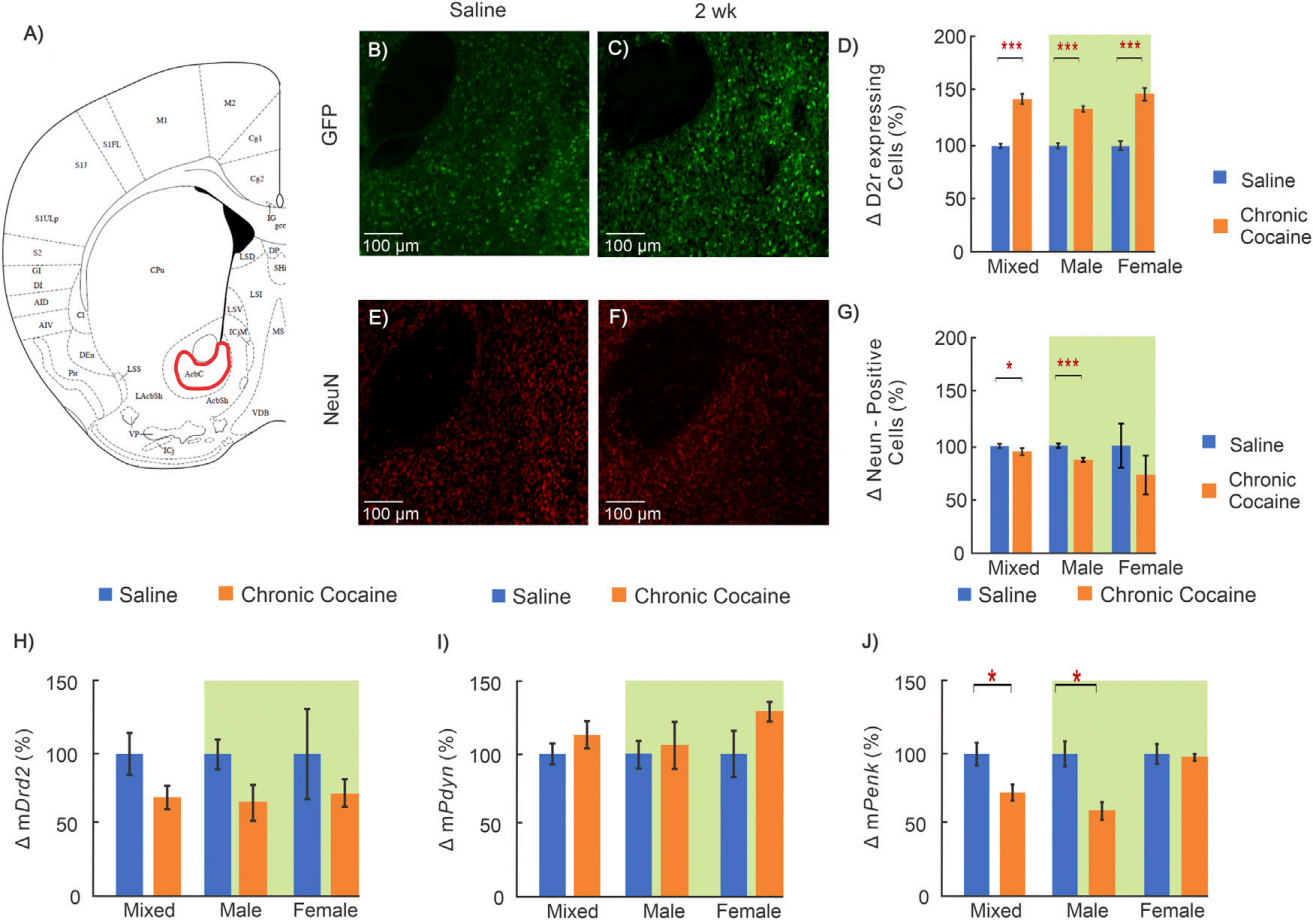
Effects of cocaine in nucleus accumbens (NAc). **(A)**: Brain atlas of the location in NAc where images were obtained (George and Franklin, 2001); **(B, E)**: “Zoom-in” GFP and NeuN sections in NAc for a representative control; and **(C, F)**: for a representative cocaine mouse; **(D)** Results comparing D2r‐GFP expressing neurons in NAc for saline (blue, n = 12; 5 ♀, 7 ♂) and cocaine exposed mice (orange, n = 11; 6 ♀, 5 ♂); and **(G)**: results for differences in total neurons (saline = 11; 5 ♀, 6 ♂, cocaine = 10; 6 ♀, 4 ♂); **(H–J)**: Results in WT mice comparing mRNA levels for **(H)**: *Drd2*, **(I)**: *Pdyn* and **(J)**: *Penk* between saline (blue, n = 12; 6 ♀,6 ♂) and cocaine treated (orange, n = 14; 7 ♀,7 ♂) animals. Mixed: including both male of female; female: female animal only; male: male only; Two-way ANOVA data indicated with green background; *: *p* < 0.05; **: *p* < 0.005; ***: *p* < 0.001.

**FIGURE 6:**
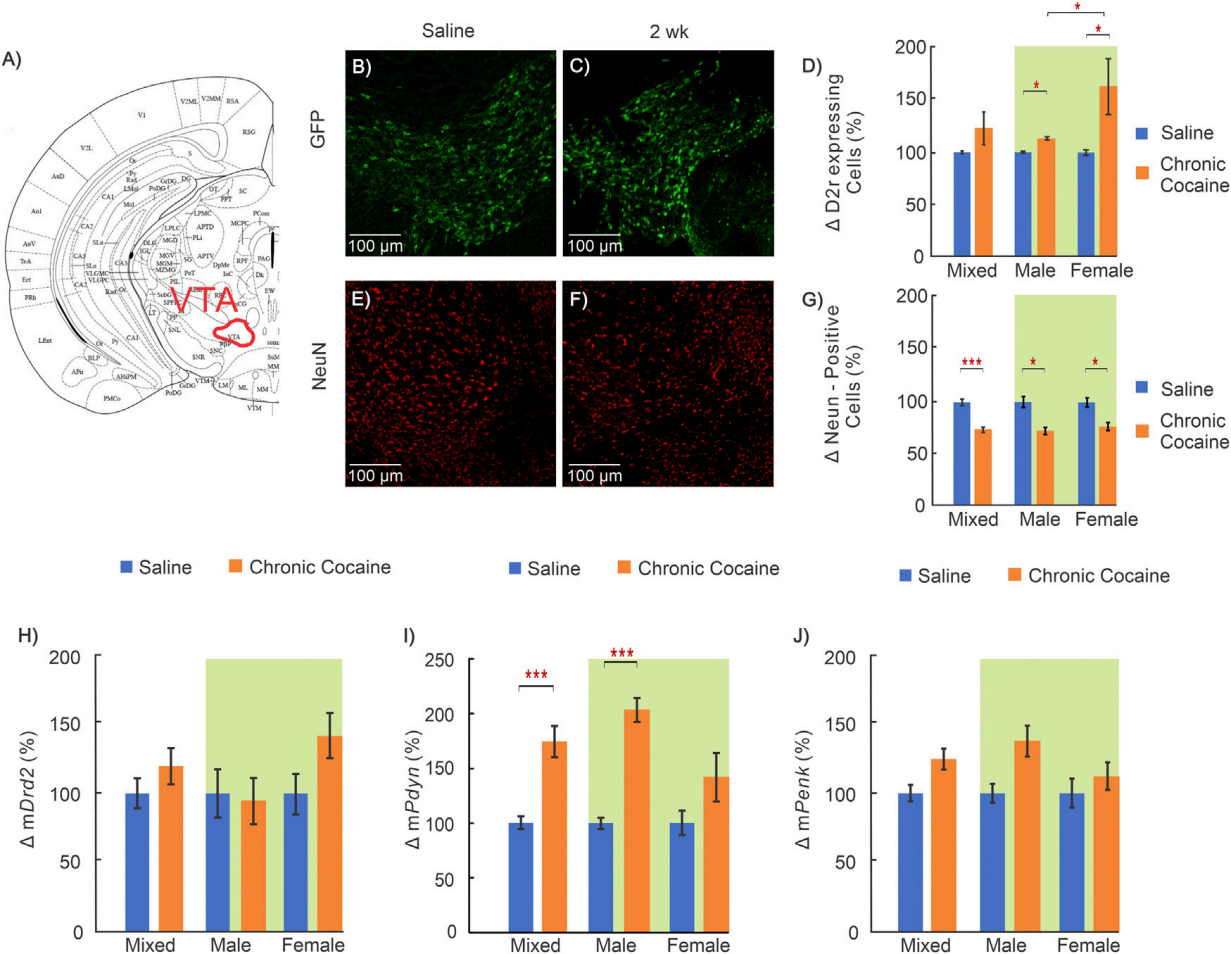
Effects of cocaine in the ventral tegmental area (VTA) **(A)**: Mouse brain atlas showing location of VTA where images were obtained (George and Franklin, 2001); **(B—C)**: “Zoom-in” GFP representative images in a control and **(E–F)** for a cocaine exposed animal for D2r‐GFP and NeuN respectively; **(D)**: Comparison of D2r‐GFP expressing neurons between saline (blue, n = 11; 5 ♀, 6 ♂) and cocaine animals (orange, n = 11; 6 ♀, 5 ♂); **(G)**: Comparison of total neuronal population between saline (n = 9; 4 ♀, 5 ♂) and cocaine (n = 10; 5 ♀, 5 ♂) exposed animals; **(H–J)**: Comparison of mRNA levels between control (n = 12; 6 ♀, 6 ♂) and cocaine (n = 14; 7 ♀, 7 ♂) animals for *Drd2* **(H)**, *Pdyn* **(I)**, and *Penk* **(J)**. Mixed: males and females; female: females only; male: males only; Data compared with two-way ANOVA indicated by green background; *: *p* < 0.05; **: *p* < 0.005; ***: *p* < 0.001.

Mice were given a lethal dose of anesthesia and then perfused transcardially with cold 1x phosphate buffered saline (PBS) followed by 4% paraformaldehyde (PFA). The brain tissue was collected and immersed in 4% PFA overnight at 4°C for further fixation. After 24 h in PFA, the tissue was transferred to a 30% sucrose solution in 1x PBS to cryoprotect it. The brain was then frozen at −80°C until sectioning. Similar to prior investigations into D2r expression, sections with a thickness of 50 μm were collected in mPFC, Dstr, NAc and VTA (Figures 2–6 provide location of sampling) (Lawhorn et al., 2013; Cheng et al., 2017; Gagnon et al., 2017; Wei et al., 2018; Dobbs et al., 2019). These sections were free floated and transferred to slides prior to staining. Slides were either stained on the same day (for Group 1B, Group 3, Group 4B, and Group 6 in Table 1) or frozen at −80°C for imaging without staining (Group 1A and Group 4A).

The Tg(Drd2-eGFP)S118Gsat mice were divided into two groups, one received saline (n = 19; Table 1: Group 1A,B) and the other cocaine (n = 23; Table 1: Group 4A,B). Each group comprised two subgroups, one (Group 1A and 4A in Table 1) was used to measure D2r fluorescence intensity in the striatum and the other (Group 1B and Group 4B in Table 1) was used for GFP and NeuN staining. For immunohistochemistry, we first washed the slides 3 times with 1x PBS followed by blocking with a solution containing 4% donkey serum, 0.03% Triton-X and 30 mg/ml of Bovine Serum Albumin (BSA) in 1x PBS. After blocking at room temperature for 1 h, slides were washed 5 times and the primary antibody solution containing 4% donkey serum, 0.03% Triton-X, chicken anti-GFP antibody (1:200, Thermofischer), and mouse anti-Neun antibody (1:200, Millpore) added. The slides were incubated in the solution at 4°C overnight on a shaker at 100 rpm. Prior to adding the secondary antibody solution, the slides were washed 6 times with 1x PBS followed by addition of the secondary antibody. This solution included 4% donkey serum, 0.03% Triton-X, Alexa-fluro 488 donkey anti-chicken (1:200, Jackson Immunoresearch), and Alex-fluro 594 donkey anti-mouse (1:200, Jackson Immunoresearch). After incubating in the secondary antibody at 4°C overnight on a shaker, the slides were washed 6 times with 1x PBS and a mounting media containing DAPi added. Slides were then cover slipped and imaged on a fluorescent microscope.

We chose NeuN over other neuronal CNS markers, such as synaptophysin, to quantify the neuronal population for it localizes only in the nuclei of neurons and not in glia and its nuclear location allows easy segmentation and quantification of neuronal density (Gusel’nikova and Korzhevskiy, 2015).

To detect D2r expression in C57BL/6 mice (Table 1: Group 3 and 6), the same protocol as outlined above for immunohistochemistry was utilized with the primary antibody rabbit anti-Drd2 (1:200, Millipore Sigma, AB5084P) and the secondary antibody Alex-fluro 594 donkey anti-rabbit (1:200, Jackson Immunoresearch).

### Fluorescent Microscopy and Image Analysis

Images were captured on a Nikon fluorescent microscope under white light at 4x to evaluate the A/P location of the section followed by imaging at 10X for each brain region. Accurate representation was achieved by nine random single focal images for each condition via different animals. Specifically, for each animal we created a mosaic of the brain region for three different brain sections. Each mosaic was composed of multiple images (∼40, as illustrated in Figure 1A) and montaged together to compose a large field of view image while retaining resolution for cell counting. In total, we obtained from each animal 12 sets of images, 3 for mPFC, 3 for Dstr, 3 for NAc and 3 for VTA with an average image dimension of 1.5 mm × 1.2 mm x 0.05 mm (length x width x depth).

Density of cells in each image was quantified using a custom ImageJ (NIH) macro to count the number of cells in the brain region. For each brain section, we quantified 3 ROI. Therefore, for a brain region (i.e., mPFC) with 14 control mice, a total of 126 ROIs were analyzed (3 ROI/section * 3 sections/animal * 14 animals = 126). Meanwhile, a total of 117 ROI were analyzed for the mixed mPFC cocaine group (*n* = 17; 3 ROI/section * 3 sections/animal * 17 animals = 153). The cell number from the macro was normalized to the volume of the brain region quantified to compute cell density. Once the cell density for each brain region was obtained, we averaged them to calculate the cell density for each specific region. Data are presented as the percent change in cell density relative to the saline controls for a specific group (i.e. percent change in D2r cell density in male cocaine exposed mice is relative to D2r cell density in saline exposed male mice).

Images for quantifying D2r-eGFP signaling without immunohistochemistry were capture on a Nikon fluorescent microscope at 10X. In total, 25 images from 6 control animals and 18 images from 5 cocaine animals were obtained from the dorsal striatum. The signal to noise ratio (SNR) was calculated by measuring the average intensity of GFP positive cells relative to background fluorescence intensity.

### RNA Extraction

Two groups of C57BL/6 background mice were used for molecular analysis, half received saline (10cc/kg, i.p. daily for 14 days; Group 2 in Table 1), and the other cocaine (30 mg/kg/day, i.p. daily for 14 days; Group 5 in Table 1). Twenty-four hours after completion of the 14 days treatment period, animals were deeply anesthetized with isoflurane and decapitated. The brain tissue was then collected from mPFC, Dstr, NAc and VTA through manual dissection using a mouse brain atlas to ensure accurate and consistent dissection. The brain tissue samples were frozen at −80°C for 5 h and shipped overnight on dry ice to Dr. Lin’s laboratory for RNA analysis. Total RNA was isolated by using TRIzol reagents (Ambion, MA, United States) following the manufacturer’s protocol. 30 μl RNase-free water was used for reconstitution. RNA concentration was determined with NanoDrop Lit (Thermoscientific, MA, United States). Usually 3 μg RNA could be extracted from 3 mg of mouse brain tissue. Samples were stored at −80°C till cDNA synthesis.

### cDNA Synthesis

100 ng RNA was reverse transcribed into cDNA using a Verso cDNA synthesis kit (Thermoscientific, MA, United States) with oligo dT primers following the manufacturer’s protocol. cDNA was diluted by 10 folds with DNase-free water for mRNA quantification or stored at −20°C.

### Quantitative Reverse Transcription Polymerase Chain Reaction (qRT-PCR) and Quantification of Relative mRNA Levels

All qRT-PCR primers used in this study were designed as intron-spanning. Two pairs of primers were designed for each gene so that the one with better performance, that is, single peak in melting curve and/or lower Ct value, was selected for estimation of its amplification coefficient (AC). Those selected primers are listed in Table 2.

**TABLE 2:** qRT-PCR primers validated, selected and used in this study.

Primer	Sequence	Tm (GC + AT)	AC
mPdyn1f	CAG​GAC​CTG​GTG​CCG​CCC​TCA​GAG	82°C	1.960
mPdyn1r	CGCTTCTGGTTGTCC CACTTCAGC	76°C	
mDrd2f	GCA​TGG​CTG​TAT​CCA​GAG​AG	62°C	1.978
mDrd2r	CCC​ACC​ACC​TCC​AGA​TAG​AC	64°C	
mPenk1f	CGA​CAT​CAA​TTT​CCT​GGC​GT	60°C	1.954
mPenk1r	AGA​TCC​TTG​CAG​GTC​TCC​CA	62°C	
mGapdhf	AGGCCGGATGTGTTCG	52°C	2.017
mGapdhr	TTA​CCA​GAG​TTA​AAA​GCA​GCC	60°C	

Samples were amplified in triple and incubated in the Bio-Rad CFX Connect real-time system (Bio-rad, CA, United States). The qRT-PCR condition was 95°C for 30 s, then for 40 cycles of 95°C for 10 s, 55°C for 20 s and 72°C for 30 s using Ssoadvanced universal SYBR Green supermix (Bio-rad, 172–5271, CA, United States) in a final volume of 12.5 μL, containing 2 μL of diluted cDNA and a final concentration of 0.5 μmol/L for forward and reverse primers. To estimate an AC, 1:2 serial dilutions of a starting cDNA sample prepared 8 cDNA concentrations, and the Ct vs log cDNA concentration plot, or standard curve, was constructed to calculate the Ct slope. AC was calculated from the Ct slope of the standard curve using the formula: AC = 10^−1/slope^. This AC was used in data analysis for relative mRNA levels. Standard curve was generated by using the same reaction system and qRT-PCR condition as for samples. Data were normalized with respect to the reference gene *Gapdh*. Results are presented as percent change relative to saline control.

### Statistics

The mean value, standard deviation, and standard error of the experimental results obtained from the immunochemistry, fluorescence imaging and qRT-PCR were calculated using Microsoft Excel. Statistics were carried out using SPSS (IBM). A three factor ANOVA was used to identify interactions between sex, treatment, and brain region and a *p*-value less than 0.05 was considered significant. Student T-tests were initially used to compare the differences in the “Mixed” groups (combined males and females) between controls and cocaine-exposed mice as the two groups only differ by one factor (treatment). If the *t*-test yielded a significant outcome for the whole group, we then investigated how each sex contributed to the outcome of the mixed group through two-way ANOVAs. Male and female data in each brain region were compared using a two factor (sex and treatment) ANOVA. When significant interactions or main effects were found, pairwise comparisons were used with a Bonferroni corrected *p*-value of less than 0.0167 indicating significance.

## Results

To assess whether chronic cocaine exposure induces alterations at the tissue and neuronal levels, we combined a D2r-eGFP transgenic mouse model with immunostaining fluorescence techniques. Immunohistochemistry was used to enhance GFP visualization of D2r-positive cells in order to count them and the neuronal nuclei indicator NeuN was used to quantify the total neuronal population. The single-cell resolution of microscope imaging enabled us to define the density of D2r-GFP+ (positive) neurons (D2r-expressing neurons) and the total density for all neurons. Comparisons between control and cocaine treated animals allowed us to assess changes from chronic cocaine exposure in the 4 brain regions.

### Interaction Between Treatment, Brain Region and Sex

Three-way ANOVAs of sex, treatment, and brain regional effects were conducted to identify potential interplay between these variables and are summarized in Table 3. A significant three-way interaction among the variables was revealed for neuronal (NeuN) density (F_(3,65)_ = 5.757, *p* = 0.001) and *Pdyn* mRNA levels (F_(3,88)_ = 3.791, *p* = 0.013) but not for D2r-GFP fluorescence, m*Drd2* or m*Penk*. Significant treatment x region interaction was observed for NeuN (*p* = 0.009) and for *Pdyn* (*p* = 0.01) and *Penk* (*p* = 0.017) mRNA levels, but not for D2r-GFP or m*Drd2*. Significant treatment x sex interaction was found for both D2r-GFP (*p* = 0.007) and *Drd2* mRNA (*p* = 0.00036), along with *Penk* mRNA (*p* = 0.026), but not for NeuN or *Pdyn* mRNA. Sex-region interactions were observed for NeuN and *Pdyn* mRNA only. In the three-way ANOVA with the independent variables sex, treatment, and brain region, significant treatment effects were observed on NeuN (*p* = 1.5 × 10^−16^), D2r-GFP (*p* = 5.1 × 10^−10^) and m*Pdyn* (*p* = 1.4 × 10^−5^); significant sex effects were recognized on both D2r-GFP (*p* = 0.002) and *Drd2* mRNA levels (*p* = 0.00036), also for m*Penk* (*p* = 0.025) but not for NeuN or m*Pdyn*. Among all the genes examined, *Drd2* showed the most significant interactions between treatment and sex. To evaluate a treatment-sex interaction in a particular brain region, two-way ANOVA was performed in the following analyses.

**TABLE 3:** Outcome of 3-way ANOVA.

	NeuN	D2r-eGFP	m*Drd2*	m*PDyn*	m*Penk*
Treatment	*p* = 1.50 × 10^−16^	*p* = 5.10 × 10^−10^	*p* = 0.732	*p* = 1.4 × 10^−5^	*p* = 0.06
Sex	*p* = 0.077	*p* = 0.002	*p* = 3.64 × 10^−4^	*p* = 0.253	*p* = 0.025
Brain region	*p* = 0.009	*p* = 0.656	*p* = 0.121	*p* = 0.01	*p* = 0.017
Sex x brain region	*p* = 0.001	*p* = 0.784	*p* = 0.103	*p* = 0.013	*p* = 0.079
Brain region x treatment	*p* = 0.009	*p* = 0.883	*p* = 0.12	*p* = 0.01	*p* = 0.017
Sex x treatment	*p* = 0.077	*p* = 0.007	*p* = 3.64 × 10^−4^	*p* = 0.253	*p* = 0.026
Treatment x sex x brain region	*p* = 0.001	*p* = 0.604	*p* = 0.103	*p* = 0.013	*p* = 0.079

### Chronic Cocaine’s Effect on the Medial Prefrontal Cortex (mPFC)

Representative images from a control mouse used to quantify D2r-eGFP expressing neurons (green cells) and the total neuronal population (red cells) are shown in Figures 2B,E, respectively, and those in a cocaine treated animal are shown in Figures 2C,F, respectively. The differences in the density of D2r-expressing neurons and of total neuron numbers between control and cocaine treated animals are summarized in Figures 2D,G. Specifically, when males and females are grouped together (i.e., defined as ‘mixed’ shown in Figure 2D), the density of D2r-expressing neurons was 45 ± 10% higher in cocaine treated than in controls (n_saline_ = 14, n_cocaine_ = 13, *p* = 0.003). A two-way ANOVA of sex and treatment revealed a significant main effect of treatment on D2r-expressing neuron density too (F_(1,23)_ = 9.82, *p* = 0.005). Pairwise comparison tests showed that increases in D2r-eGFP cell density were significant only in females (i.e. Females: 60 ± 12%, n_saline_ = 5, n_cocaine_ = 6, *p* = 0.016), but not males (Males: 38 ± 17%, n_saline_ = 9, n_cocaine_ = 7, *p* = 0.08). In contrast, the number of NeuN positive cells (reflecting total neuron count) in mPFC was 35 ± 6% lower (mixed group) in cocaine exposed animals compared to controls (n_saline_ = 10, n_cocaine_ = 10, *p* < 0.001). A two-way ANOVA for NeuN density found a significant interaction between sex and treatment (F_(1,17)_ = 17.28, *p* < 0.001). Pairwise comparison of neuronal density showed that the density was lower in both male (Males: 16 ± 5% decrease, n_saline_ = 5, n_cocaine_ = 4, *p* = 0.01) and females (47 ± 5% decrease, n_saline_ = 5, n_cocaine_ = 6, *p* < 0.001). The decrease in neuronal density with cocaine was larger in females than in males (*p* < 0.001).

Receptor transcription levels were evaluated using qRT-PCR to measure mRNA of *Drd2*, *Pdyn*, and *Penk* in mPFC. Two way ANOVA for m*Drd2* demonstrated a significant interaction between sex and treatment (F_(1,22)_ = 8.74, *p* = 0.007). Pairwise comparison revealed that the mRNA for *Drd2* in female mice exposed to cocaine was greater than control (76 ± 18%; n_saline_ = 6, n_cocaine_ = 7, *p* = 0.016) and was significantly larger than m*Drd2* in cocaine treated males (*p* = 3.1 × 10^−4^). Similar to m*Drd2*, two-way ANOVA of m*Penk* revealed a significant interaction between sex and treatment (F_(1,22)_ = 4.37, *p* = 0.048) with pairwise comparison demonstrating that after cocaine, female m*Penk* levels were significantly higher than female saline control (81 ± 19%; n_saline_ = 6, n_cocaine_ = 7, *p* = 0.01), and male cocaine treated mice (n_cocaine, male_ = 7, n_cocaine, female_ = 7, *p* = 0.007). (Figures 2H-J).

### Chronic Cocaine’s Effect on the Dorsal Striatum (Dstr)

The Dstr is implicated in the neuroadaptations associated with the transition from controlled to habitual cocaine intake (Everitt et al., 2008; Koob and Volkow, 2010). Figure 3 summarizes the cocaine-associated changes in Dstr. A significant interaction between sex and treatment remained for the density of D2r-expressing neurons in the Dstr when a two-way ANOVA was performed (F_(1,19)_ = 8.07, *p* = 0.01). Pairwise analysis found that both cocaine exposed males (32 ± 4% increase; n_saline_ = 7, n_cocaine_ = 6, *p* < 0.001) and females (63 ± 11% increase; n_saline_ = 5, n_cocaine_ = 5, *p* < 0.001) showed significantly higher density of D2r-expressing neurons in Dstr than controls, with female mice having the greatest increases (n_cocaine, male_ = 6, n_cocaine, female_ = 5,*p* = 0.013) (Figure 3D). The density of neurons labeled by NeuN was analyzed using a two-way ANOVA which revealed a significant main effect of treatment (F_(1,17)_ = 18.93, *p* < 0.001). Pairwise comparisons found that female mice exposed to cocaine had significantly lower neuronal density than female saline controls(24 ± 6% decrease; n_saline_ = 5, n_cocaine_ = 6, *p* = 0.006) (Figure 3G).

Two-way ANOVA was run for *Drd2, Penk,* and *Pdyn* mRNA levels in the Dstr. The ANOVA showed a significant interaction between sex and treatment for m*Drd2* (F_(1,22)_ = 8.25, *p* = 0.009) but not for m*Penk* or m*Pdyn*. Pairwise analysis determined that in cocaine treated female mice, the level of m*Drd2* was significantly higher compared to female controls (53 ± 14% increase; n_saline_ = 6, n_cocaine_ = 7, *p* = 0.016) whereas males showed a non-significant decrease (26%; n_saline_ = 6, n_cocaine_ = 7, *p* = 0.2) (Figure 3H).

Using immunohistochemistry, we observed an increase in the number of D2r-positive neurons in mPFC and Dstr in mice exposed to cocaine. To confirm this, we utilized an antibody that binds to the D2r protein, which we measured in the dorsal striatum of WT animals to quantify differences between saline and cocaine treated mice. Figure 4F,G show representative images of D2r positive cell density in the dorsal striatum of WT mice exposed to saline and cocaine pretreatment. Comparison of the density of D2r-positive cells between saline (n = 3) and cocaine treated (n = 3) C57BL/6 mice showed ∼ 36% increase in the cocaine exposed group compared to controls (*p* = 0.02, Figure 4H); which is in close agreement to the results observed in the D2r-eGFP mice (Figures 3B–D).

To assess the effects of cocaine on *Drd2* expression levels at the neuronal level. we used D2r-GFP fluorescence intensity as a marker of *Drd2* expression in Dstr neurons. Prior studies in BAC transgenic mice showed an association between fluorescence intensity (no immunohistochemistry) and gene expression (Zhou et al., 2011; Crook and Housman, 2012; Lawhorn et al., 2013). To confirm that the GFP observed in the D2r-eGFP reporter line corresponded to D2r expression we assessed the co-localization of the D2r-eGFP signal with that of the D2r antibody in the dorsal striatum. Figure 4B-D show the representative images from a mouse’s brain of the co-localization of GFP from D2r-eGFP reporter line (green), D2r antibody (red), and merged (yellow). The overlap between eGFP and D2r staining was 86 ± 7% (n = 3), indicating that D2r-eGFP represents cells expressing D2r. The comparison of D2r intensity between controls (Table 1: Group 1A) and cocaine-exposed mice (Table 1: Group 4A) is summarized in Figure 4I-K. Cocaine exposed mice showed a decrease in normalized GFP fluorescence, i.e., ratio of GFP fluorescence over the background (ΔGFP-SNR = 0.44 ± 0.10 decrease; n_saline_ = 5, n_cocaine_ = 6, *p* = 0.002) relative to controls (Figure 4K).

Taken together these results suggests that cocaine increased the number of D2r-expressing neurons amid an associated reduction of the total neuronal population. Findings also suggest that the level of expression of the *Drd2* gene in D2r-expressing neurons, as evidence by decreased fluorescence intensity, was lower in cocaine exposed mice than in controls.

### Chronic Cocaine’s Effect on the Nucleus Accumbens (NAc)

A two-way ANOVA for sex and treatment was conducted on D2r-expressing neurons in the NAc. The ANOVA revealed a significant interaction between sex and treatment (F_(1,19)_ = 6.621, *p* = 0.019) for this brain region. Pairwise comparison showed the number of D2r-expressing neurons increased in both sexes though females had a 96% greater increase than males (Males: 24 ± 3%, n_saline_ = 7, n_cocaine_ = 5, *p* < 0.001; Females: 47 ± 6%, n_saline_ = 5, n_cocaine_ = 6, *p* < 0.001; Male cocaine v female cocaine *p* = 0.014) (Figure 5D). Two way ANOVA for NeuN-positive cells showed a main effect of treatment (F_(1,17)_ = 15.87, *p* < 0.001) with pairwise analysis revealing that D2r-expressing neurons in male cocaine-exposed mice were decreased by 20 ± 2% (n_saline_ = 6, n_cocaine_ = 4, *p* < 0.001) (Figure 5G).

*Drd2* mRNA levels did not show a treatment effect neither in male nor female mice (Figure 5H). For *Penk* mRNA levels there was a significant effect of treatment (F_(1,22)_ = 4.86, *p* = 0.038). In cocaine treated males m*Penk* levels were decreased by 42 ± 8% (n_saline_ = 6, n_cocaine_ = 7, *p* = 0.007) whereas females showed no changes (3 ± 3% NS) (Figure 5J). The *Pdyn* mRNA levels did not show a treatment effect neither in male nor female mice (Figure 5I).

### Chronic Cocaine’s Effect on the Ventral Tegmental Area (VTA)

In the VTA, which is the location of dopamine neurons that project to NAc, the number of D2r-positive cells was increased in cocaine treated mice compared to controls. Two-way ANOVA revealed a significant main effect of treatment (F_(1,17)_ = 5.51, *p* = 0.031) with pairwise comparisons demonstrating both male and female mice had a significant increase in the density of D2r- neurons following cocaine exposure (Males: 9 ± 3%, n_saline_ = 6, n_cocaine_ = 5, *p* = 0.016; Females: 64 ± 3%, n_saline_ = 5, n_cocaine_ = 6, *p* = 0.008) (Figure 6D). Similarly, two-way ANOVA for NeuN revealed a significant main effect of treatment. Conversely to GFP, pairwise comparison found NeuN positive cells were decreased by 27 ± 4% (n_saline_ = 5, n_cocaine_ = 5, *p* = 0.005) in males and 25 ± 4% (n_saline_ = 5, n_cocaine_ = 5, *p* = 0.006) in female cocaine-expose mice compared to sex matched saline-exposed mice (Figure 6G). The mRNA for *Drd2* and *Penk* did not differ between groups (Figures 6H,J). Analysis of *Pdyn* mRNA results using two-way ANOVA demonstrated significant effect of treatment (F_(1,22)_ = 24.38, *p* < 0.001). Pairwise comparisons found m*Pdyn* was significantly increased in cocaine-exposed male mice by 117 ± 13% (n_saline_ = 6, n_cocaine_ = 7, *p* < 0.001) (Figure 6I).

## Discussion

Here we assessed the effects of chronic cocaine (2 weeks passive exposure) on D2r-expressing neurons and the total neuronal population in the dopamine mesocortical system. Based on findings reporting decreased D2r availability in striatum we expected to observe a reduction in D2r-expressing neurons. However, contrary to our hypothesis, we found a significant increase in the number of D2r-expressing neurons in mPFC, Dstr, NAc, and VTA in cocaine exposed mice that was stronger in females than males. These increases occurred amidst decreases in the total number of neurons in mPFC and Dstr and these changes were also larger in cocaine exposed females than males. Cocaine exposed females also showed increases in *Drd2* mRNA levels in mPFC and Dstr, consistent with the findings of increases in D2r-expressing neurons, whereas in males there were no significant changes in *Drd2* mRNA. Similarly, whereas m*Penk* were increased in cocaine exposed females in mPFC; in cocaine-exposed males m*Pdyn* was increased in VTA and m*Penk* was decreased in NAc compared to male saline controls.

To our knowledge, this is the first report of an upregulation in the number of neurons that express *Drd2* in the dopamine mesocortical system in animals chronically exposed to cocaine. The increases in D2r-expressing neurons was larger in mPFC and VTA of females exposed to cocaine than in males. Though in females there were parallel increases in *Drd2* mRNA in mPFC and Dstr, this was not the case for NAc or VTA nor was it observed in males. The reason for the discrepancies between the increases in the number of D2r-expressing neurons and the lack of changes in mRNA *Drd2* levels in the VTA and NAc of both sexes and in the Dstr of males is unclear. This divergence in the results of the two methodologies indicates that the increase in the number of D2r-expressing neurons was not reflected in the *Drd2* expression measures averaged at the tissue level. In other words, the changes in expression of *Drd2* as reflected by the number of cells that expressed eGFP regulated by the *Drd2* promoter provided different results from the measures obtained when averaging mRNA levels across all cells in the tissue*.* Such a discrepancy could reflect the fact that with eGFP transcription of the *Drd2* gene, even if it happened at a very low level, it will lead to fluorescence of the neuron making it detectable whereas in the tissue the varying levels of mRNA would be averaged across all cells diluting the signal.

We interpret the increase in D2r-expressing neurons amidst a decrease in the total number of neurons to reflect a phenotypic shift in some of the surviving neurons that result in activation of a previously silent *Drd2* gene. Indeed, reports have shown that striatal neurons can undergo phenotypic shifts when dopamine (DA) neurons are damaged or activated. For example, Tande et al. reported a 2-fold increase in the number of striatal neurons expressing the dopamine transporter in macaques exposed to MPTP (1-methyl-4-phenyl-1,2,3,6-tetrahydropyridine) that reflected a phenotypic shift of pre-existing small spiny GABAergic interneurons (Tandé et al., 2006). In addition, Dela Cruz et al. reported an increase in the number of tyrosine hydroxylase immunoreactive neurons in the VTA after deep brain stimulation (Dela Cruz et al., 2015). These studies provide supporting evidence that changes in dopamine signaling can trigger phenotypic neuronal transdifferentiation within the mesocortical dopamine system. To our knowledge, our study is the first to document an increase in the number of D2r expressing neurons following chronic cocaine exposure. However, increases in the number of neurons expressing a particular molecule are not unique to cocaine or the dopamine mesocortical system. Specifically, a recent histochemistry study reported that chronic morphine increased the number of hypothalamic neurons that expressed hypocretin that was not due to neurogenesis and this finding was corroborated in postmortem brains of heroin users (Thannickal et al., 2018). Evidence for shifts in neuronal phenotypes is considered by some to be another form of neuroplasticity (Dulcis et al., 2017). Future studies are needed to determine if the increases in the number of D2r expressing neurons with chronic cocaine reflects transdifferentiation and if so, to identify the neuronal types within the mesocortical system that activate a previously silent *Drd2* gene and the mechanisms that drive it.

Cocaine mediates its effects by inhibiting the dopamine transporter thus increasing extracellular DA. Increases in DA levels underlie cocaine’s rewarding effects but have also been implicated in excitotoxicity through enhanced glutamatergic stimulation (Kerkerian et al., 1987; Olney et al., 1991; Dauer and Przedborski, 2003) including NMDA receptor mediated cell swelling (Dodt et al., 1993; Colwell and Levine, 1996). Studies have shown that D2r activation reduces NMDA excitotoxic cell swelling and triggers internalization of AMPA glutamate receptors (Cepeda et al., 1998; Zou et al., 2005) supporting a role of D2r in mitigating excitotoxicity. Therefore, our observation of an increase in the density of D2r-postive cells may be a compensatory mechanism to reduce excitotoxic effects of cocaine-induced DA increases and presumably also to decrease DA release through overexpression of D2r auto-receptors. The neuronal loss reported in our investigation may reflect a failure of this compensatory mechanism.

The sex differences in response to cocaine could reflect the previously reported dimorphism in synapse and neuronal function in brain reward regions. Specifically, Wissman et al. using voltage clamp recording of neurons in the NAc revealed that female rats have higher basal miniature excitatory postsynaptic potential (mEPSC) than males in the NAc core but not shell, and chronic cocaine increased mEPSC to a greater extent in females than males (Wissman et al., 2011). Sex hormones may underlie some of these differences since they influence basal neuronal activity and synaptic DA kinetics. Estrus females have increased basal VTA activity as compared to non-estrus and male mice and release two times more DA into the synapse with tonic stimulation and display impaired synaptic D2-autoreceptor function (Calipari et al., 2017).

In contrast we observed a decrease in D2r-eGFP fluorescence intensity across the Dstr of cocaine exposed mice, which we interpret to reflect a reduction in the overall expression of D2r in the tissue (Zhou et al., 2011; Crook and Housman, 2012). The increase in the number of D2r-expressing neurons with a simultaneous reduced fluorescence intensity in the tissue (measured only in Dstr) suggests that while more neurons expressed *Drd2* those neurons might have had lower *Drd2* expression levels. Alternatively, this apparent discrepancy could also reflect a redistribution of fluorescence in D2r expressing neurons in cocaine exposed mice if a phenotypic shift in expression of D2r occurred in small interneurons instead of the typical D2r expression in striatal MSN. Indeed in the MPTP model of Parkinson’s disease, an increase in tyrosine hydroxylase and DAT expressing neurons in the striatum occurred by the phenotypic transformation of small interneurons (Tandé et al., 2006). Regardless, such a marked disruption of D2r expression could unbalance DA signaling in the reward pathway contributing to compulsive cocaine seeking as observed with optogenetic inhibition of D2r-expressing neurons (Baik, 2013; Song et al., 2014).

The dopamine receptors D1r and D2r exert opposite effects on neuronal excitability with D1r increasing and D2r decreasing it (Alvarez, 2016). D1r and D2r expressing neurons in the striatum also have distinct expression patterns for endogenous opioids; enkephalin is mainly co-expressed in D2r whereas dynorphin is mainly co-expressed in D1r-expressing neurons (Surmeier et al., 1998; Gerfen and Surmeier, 2011; Gangarossa et al., 2013). Thus, we also quantified mRNA for proenkephalin (m*Penk*) and prodynorphin (m*Pdyn*). We measured the mRNA levels of m*Pdyn* as a control since it is expressed in D1r- expressing cells to contrast it with the changes in mRNA levels in D2r and m*Penk*, which are expressed in D2r-expressing cells. Females exposed to chronic cocaine only showed elevated levels of m*Penk* in the PFC whereas males showed decreases in m*Penk* in NAc and increases in m*Pdyn* in VTA. Dynorphin, the protein product of m*Pdyn*, reduces firing of DA neurons in the VTA (Muschamp et al., 2014; Baimel et al., 2017). The upregulation of dynorphin in the VTA suggest that chronic cocaine exposure might lead to a reduction in DA neuronal firing and in DA release.

After chronic cocaine we observed a decrease in the density of cells which stained positive for NeuN, which suggests neuronal loss after chronic cocaine exposure. A reduction in neuronal nuclei following cocaine exposure has been previously reported in *ex-vivo* and *in-vitro* studies and has been attributed to cocaine’s neurotoxic effects (George et al., 2008; Lepsch et al., 2015). The *in-vitro* study of Lepsch et al. reported a reduction of NeuN in cultured primary striatal neurons exposed to cocaine due to activation of apoptotic pathways (Lepsch et al., 2015) triggered by nitrosylation of glyceraldehyde-3-phosphate dehydrogenase by nitric oxide leading to activation of p53 and its downstream components PUMA and Bax (Xu et al., 2013; Guha et al., 2016). Thus, the global decrease in neurons observed in the cocaine exposed mice in our study could reflect cocaine’s apoptotic effects. In addition, both clinical and animal studies have documented cocaine induced vasoconstriction leading to cerebral ischemia in cocaine addicted individuals and in laboratory animals exposed to cocaine (Treadwell and Robinson, 2007; You et al., 2017). NeuN levels have been shown to decrease in response to ischemia and thus neuronal loss with cocaine could also reflects its vasoconstrictive effects (Gusel’nikova and Korzhevskiy, 2015).

The decrease in the neuronal population following chronic cocaine exposure was largest in mPFC and it was stronger in females than males. These findings are consistent with prior reports of neuronal loss in PFC associated with cocaine exposure in rodents and in postmortem brain studies documenting loss of DA neurons in cocaine users and neuronal loss in multiple brain regions in polysubstance users (Büttner and Weis, 2006; George et al., 2008; Little et al., 2009). They could also explain the reduction of cerebral blood flow, metabolic activity, and cortical thickness in the frontal cortex of cocaine users (Volkow et al., 1988; Hirsiger et al., 2019). Notably the study documenting loss of DA neurons in cocaine users led to the speculation of an increased risk for Parkinson’s disease in cocaine users that as of now has not been corroborated.

Our results show that the detrimental effects from cocaine exposure on the female are greater than the male brain, which are in agreement with previous reports from preclinical and clinical studies. In rodents, sex-dependent behavioral differences in response to cocaine have been observed. After passive exposure to cocaine for 9 days, cocaine treated females had greater increases in activity and rearing behaviors than cocaine treated males (van Haaren and Meyer, 1991). In self-administration experiments, female rats self-administer cocaine more times than males and also escalate drug taking faster. (Algallal et al., 2020). In humans, progression from substance use to substance dependence appears to be faster in females than males, also they appear to consume higher doses and have more difficulty in achieving and maintaining abstinence (O’Brien & Anthony, 2005; Gallop et al., 2007). Similarly, brain imaging studies of stimulant-dependent females showed that compared to males, they had larger decreases in gray matter volumes in frontal, limbic, temporal and parietal regions (Tanabe et al., 2013; Regner et al., 2015), consistent with the loss of neurons we observed in the female mice, which was most prominent in PFC. Among all the genes examined *Drd2* displayed the most significant sex effects and the most significant sex-treatment interactions, supporting a view that D2r might contribute to sex differences in cocaine behaviors.

The transgenic mouse model that we used to visualize D2r-expressing neurons was shown by a prior study to display a two-fold upregulation of *Drd2* mRNA (Kramer et al., 2011). However since we compared D2r-eGFP changes between naïve and chronic cocaine animals, any potential baseline differences in the transgenic model should not affect the comparison analyses. To confirm that the GFP in the D2r-eGFP reporter line corresponded to D2r expression, we assessed the co-localization of the D2r-eGFP signal and the D2r antibody. The high overlap (i.e., 86 ± 7%, *n* = 3) between eGFP and D2r staining indicates that D2r-eGFP is representative of cells expressing D2r.

Limitations of this study are as follows: 1) in our study we used passive cocaine administration and findings might differ with self-administering animals, which is more relevant to the clinical situation. The passive cocaine model however allowed us to remove the confounds due to dose differences; 2) we did not record behavior which would have allowed us to correlate behavioral changes with the changes in D2r expressing neurons. These would have been desirable since prior studies mostly done in males have shown that D2r availability modulates cocaine’s effects. Mice and rats with suppressed or absent D2 receptors show increased locomotion during cocaine self-administration compared to wildtypes but do not differ in the time to acquisition of cocaine self-administration (Chausmer et al., 2002; de Jong et al., 2015). When the role of D2r in cocaine relapse and seeking was studied, systemic administration of D2 agonist enhanced cocaine seeking whereas administration of the D2r antagonist raclopride into the VTA inhibited relapse to cocaine seeking (Wise et al., 1990; Xue et al., 2011); 3) we did not measure the protein levels of D2r, enkephalin and dynorphin, which would have allowed us to assess the association between the changes in mRNA and the levels of protein expression. 4) Technically, use of isoflurane in brain harvesting might systemically interfere with gene activity (Staib-Lasarzik et al., 2014; Ko et al., 2019). Additionally, while we interpret our findings of increases in D2r-expressing neurons to reflect expression of *Drd2* in neurons where the gene was previously silent, further work is needed to corroborate this. Future investigations will need to assess the consequences of these changes in brain function and behaviors relevant to cocaine addiction.

In summary, we performed a systematic assessment of the total neuronal population and of the subset of neurons expressing D2r in response to chronic cocaine using immunohistochemistry and a transgenic D2r-eGFP mouse line. This approach revealed an increase in the number of neurons expressing *Drd2* and a decrease in total neuron numbers in mPFC, Dstr, NAc, and VTA that was more severe for females than males. We interpret our findings to suggest evidence that cocaine triggers neuronal transdifferentiation in regions of the dopamine mesocortical system, but further work is needed to corroborate this.

## Data Availability

The data that support the findings of this study are available from the corresponding authors upon reasonable request.
